# Hemodynamic MRI parameters to predict asymptomatic unilateral carotid artery stenosis with random forest machine learning

**DOI:** 10.3389/fnimg.2022.1056503

**Published:** 2023-01-12

**Authors:** Carina Gleißner, Stephan Kaczmarz, Jan Kufer, Lena Schmitzer, Michael Kallmayer, Claus Zimmer, Benedikt Wiestler, Christine Preibisch, Jens Göttler

**Affiliations:** ^1^Department of Diagnostic and Interventional Neuroradiology, School of Medicine, Technical University of Munich, Munich, Germany; ^2^Philips GmbH Market DACH, Hamburg, Germany; ^3^TUM Neuroimaging Center, School of Medicine, Technical University of Munich, Munich, Germany; ^4^Department of Vascular and Endovascular Surgery, School of Medicine, Technical University of Munich, Munich, Germany; ^5^Clinic for Neurology, School of Medicine, Technical University of Munich, Munich, Germany

**Keywords:** asymptomatic carotid artery stenosis, hemodynamics, random forest–ensemble classifier, machine learning, individual watershed areas, magnetic resonance imaging (MRI)

## Abstract

**Background:**

Internal carotid artery stenosis (ICAS) can cause stroke and cognitive decline. Associated hemodynamic impairments, which are most pronounced within individual watershed areas (iWSA) between vascular territories, can be assessed with hemodynamic-oxygenation-sensitive MRI and may help to detect severely affected patients. We aimed to identify the most sensitive parameters and volumes of interest (VOI) to predict high-grade ICAS with random forest machine learning. We hypothesized an increased predictive ability considering iWSAs and a decreased cognitive performance in correctly classified patients.

**Materials and methods:**

Twenty-four patients with asymptomatic, unilateral, high-grade carotid artery stenosis and 24 age-matched healthy controls underwent MRI comprising pseudo-continuous arterial spin labeling (pCASL), breath-holding functional MRI (BH-fMRI), dynamic susceptibility contrast (DSC), T2 and T2^*^ mapping, MPRAGE and FLAIR. Quantitative maps of eight perfusion, oxygenation and microvascular parameters were obtained. Mean values of respective parameters within and outside of iWSAs split into gray (GM) and white matter (WM) were calculated for both hemispheres and for interhemispheric differences resulting in 96 features. Random forest classifiers were trained on whole GM/WM VOIs, VOIs considering iWSAs and with additional feature selection, respectively.

**Results:**

The most sensitive features in decreasing order were time-to-peak (TTP), cerebral blood flow (CBF) and cerebral vascular reactivity (CVR), all of these inside of iWSAs. Applying iWSAs combined with feature selection yielded significantly higher receiver operating characteristics areas under the curve (AUC) than whole GM/WM VOIs (AUC: 0.84 vs. 0.90, *p* = 0.039). Correctly predicted patients presented with worse cognitive performances than frequently misclassified patients (Trail-making-test B: 152.5s vs. 94.4s, *p* = 0.034).

**Conclusion:**

Random forest classifiers trained on multiparametric MRI data allow identification of the most relevant parameters and VOIs to predict ICAS, which may improve personalized treatments.

## 1. Introduction

Internal carotid artery stenosis (ICAS) is a major public health issue causing about 10% of all ischemic strokes (Flaherty et al., 2013). Furthermore, although considered clinically asymptomatic if no signs of stroke or transitory ischemic attacks can be observed, some ICAS patients develop cognitive impairments comparable to dementia (Lal et al., 2017), which might be caused by chronic cerebral hypoperfusion (Göttler et al., 2018). Efficient revascularization procedures, such as stenting or endarterectomy, are available. However, these are invasive and come with substantial periprocedural risks that must be considered carefully for asymptomatic ICAS patients, since the annual risk of stroke under best medical therapy is reported to be <1% (den Hartog et al., 2013). Consequently, the identification of severely affected patients who might benefit most from a more aggressive treatment is crucial.

In recent years, a large variety of perfusion and oxygenation sensitive MRI parameters have been suggested for the assessment of brain damage and the prediction of individual stroke risk in ICAS (Chen, 2019; Kaczmarz et al., 2021). A decrease in cerebral blood flow (CBF) (Baradaran and Gupta, 2020) derived from pseudo-continuous arterial spin labeling (pCASL) and a reduction of cerebral vascular reactivity (CVR) (King et al., 2011) obtained by breath-holding functional MRI (BH-fMRI) have been proposed as potential biomarkers to predict strokes. Furthermore, the sensitivity of time to peak (TTP), relative cerebral blood volume (rCBV), mean transit time (MTT), oxygen extraction capacity (OEC), and capillary transit-time heterogeneity (CTH) to vascular impairment has been investigated intensively using dynamic susceptibility contrast (DSC) imaging based on the injection of a gadolinium-containing tracer (Nasel et al., 2001; Mouridsen et al., 2014; Kaczmarz et al., 2021). Also, downstream alterations of the relative oxygen extraction fraction (rOEF) calculated by multi-parametric quantitative blood oxygen level dependent (mq-BOLD) MRI (Hirsch et al., 2014) have been discussed controversially (Baron et al., 1981; Chen, 2019; Göttler et al., 2019). In addition, individual watershed areas (iWSA), located at the edge of vascular territories, have been reported to be most vulnerable to these impairments (Kaczmarz et al., 2018) and are of special interest as they are also a typical location of ICAS associated strokes (Yong et al., 2006). However, it is currently unclear, which of these numerous MRI parameters are best suited to predict disease severity (Baradaran and Gupta, 2020), and whether hemodynamic or metabolic changes within iWSAs have a higher discriminative ability (Kaczmarz et al., 2021).

The identification of the most sensitive parameters and volumes of interest (VOI) to predict ICAS could provide a deeper understanding of the pathology, help to adjust treatment in an early stage of disease, and point out the most relevant parameters for further research. Additionally, it would increase the clinical applicability of hemodynamic and oxygenation sensitive MRI if the examination protocol could be restricted to the most relevant parameters, which could also be used to screen for severely affected ICAS patients.

Lately, various machine learning algorithms have been applied to neuroimaging data for disease prediction (Jollans et al., 2019). Especially the random forest classifier, which is based on an ensemble of decision trees (Breiman, 2001), has been used widely (Lebedev et al., 2014; Maggipinto et al., 2017; Carlson et al., 2020) to analyze data with respect to underlying relationships between parameters. The classifier's popularity is due to its ability to deal with high-dimensional data sets (Gregorutti et al., 2017), and its reduced tendency to overfit compared to other classification models, while maintaining high accuracies (Breiman, 2001). In addition, the importance of each variable, called feature, can be calculated enabling an embedded feature selection and a ranking of the most relevant parameters and VOIs (Breiman, 1996).

In the present study, we aimed to predict ICAS by applying a random forest classifier to an extensive set of eight multi-modal MRI parameters of a previously published study (Göttler et al., 2018, 2019; Kaczmarz et al., 2021; Schmitzer et al., 2021) that investigated hemodynamic impairments using VOIs within and outside of iWSAs (Kaczmarz et al., 2018) in asymptomatic high-grade ICAS patients and age-matched healthy controls. We hypothesize an increased accuracy when considering parameters within iWSAs. Furthermore, we analyzed the influence of the cognitive status on the patients' misclassification probabilities.

## 2. Materials and methods

### 2.1. Subjects

Twenty-nine patients (9 females, mean age 70.3 ± 7.0 years) with an asymptomatic, one-sided, high-grade extracranial ICAS [confirmed by duplex ultrasonography; all > 70% according to the NASCET criteria (NASCET, 1991)] and 30 healthy elderly (17 females, mean age 70.3 ± 4.8 years) participated in this prospective study. The study was approved by the medical ethical board of the Klinikum rechts der Isar and in line with Human Research Committee guidelines of the Technische Universität München. All participants provided informed consent in accordance with the Declaration of Helsinki. After being diagnosed with screening methods, patients were recruited in the outpatient clinic for carotid stenoses of the Department of Vascular and Endovascular Surgery and Angiology of our hospital, and healthy controls were recruited by word-of-mouth advertisement from May 2015 until May 2017. MRI neck angiographies were used to confirm the lack of stenoses in the healthy controls.

Examination of every participant included MRI, the medical history, and basic screening for neurological and psychiatric diseases. The cognitive status of the study participants was assessed by the Trail making test A and B (TMT-A/B) and Mini-Mental State Examination (MMSE). Additionally, Beck's Depression Inventory (BDI) and State Trait Anxiety Inventory (STAI) were conducted to screen for affective disorders, as these may impair cognitive performance. Data from this study cohort have been previously investigated with respect to hemodynamic impairments and variability of individual watershed areas (iWSAs) in asymptomatic ICAS (Göttler et al., 2018, 2019; Kaczmarz et al., 2018, 2021; Schmitzer et al., 2021).

Exclusion criteria for enrolment in the study were any neurological or psychiatric diseases, severe chronic kidney disease, active cancer, clinically remarkable structural MRI (e.g., territorial stroke lesions, bleedings, or a history of brain surgery), and MRI contraindications.

### 2.2. Magnetic resonance imaging and parameter calculation

MRI data were acquired on a clinical 3T Philips Ingenia MRI-Scanner (Philips Healthcare, Best, The Netherlands) using a 32- and a 16-channel head/neck-receive-coil. All MR image processing procedures used custom MATLAB programs (MATLAB R2016b, MathWorks, Natick, MA, USA) and SPM12 (Wellcome Trust Center for Neuroimaging, UCL, London, UK) and were conducted as described previously (Kaczmarz et al., 2018, 2021; Göttler et al., 2019).

**Figure 1:**
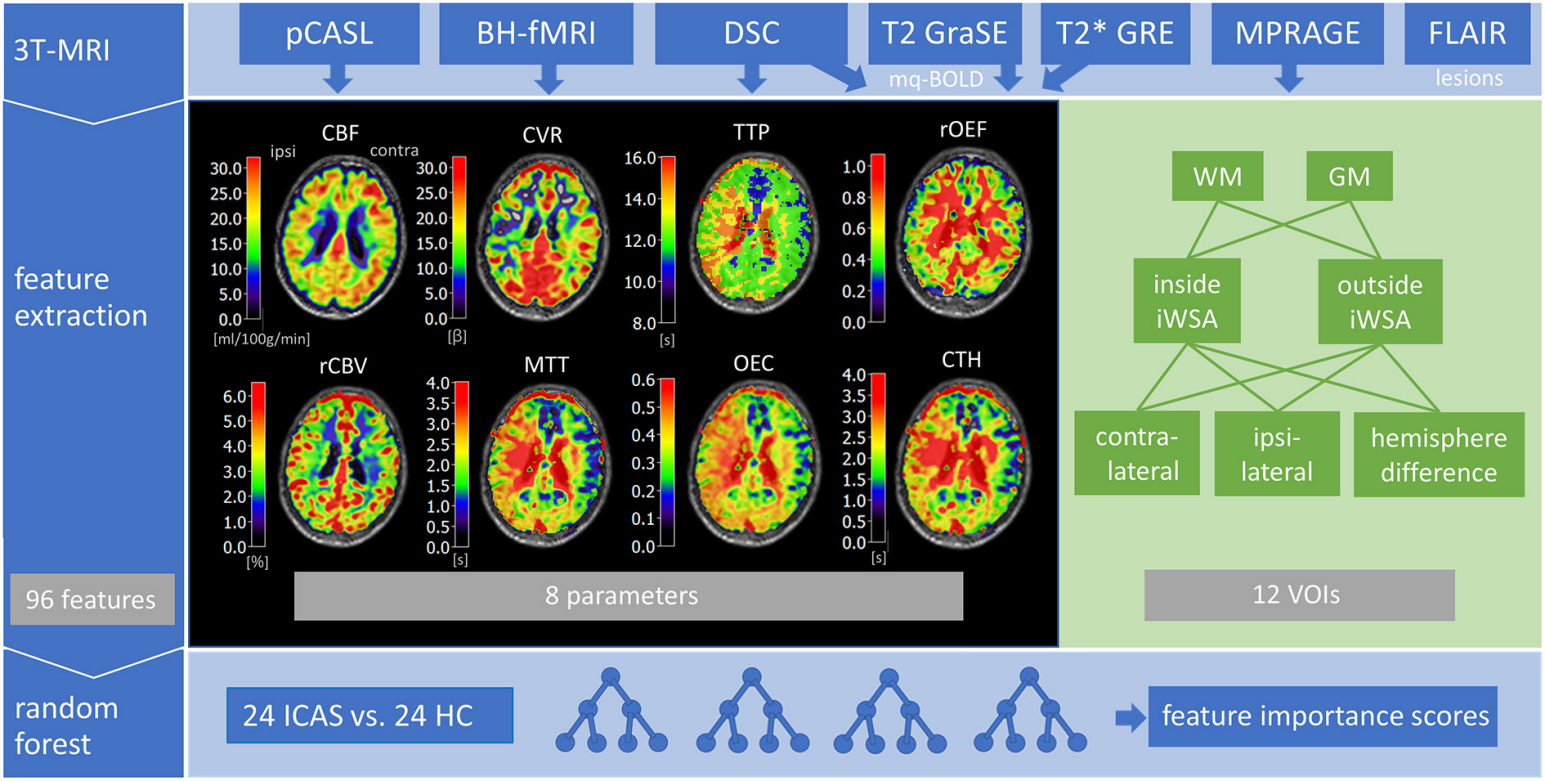
Overview of included multi-parametric MRI data and major processing steps. The 3T-MRI comprised pseudo-continuous arterial spin labeling (pCASL) to obtain cerebral blood flow (CBF), breath-holding fMRI (BH-fMRI) for cerebral vascular reactivity (CVR), dynamic susceptibility contrast (DSC) MRI to measure time to peak (TTP), relative cerebral blood volume (rCBV), mean transit time (MTT), oxygen extraction capacity (OEC), and capillary transit-time heterogeneity (CTH), FLAIR to detect lesions and MP-RAGE to generate white matter (WM) and gray matter (GM) masks. Relative oxygen extraction fraction (rOEF) was modeled from quantitative transverse relaxation times T2 and T2^*^ as well as rCBV by multi-parametric quantitative blood oxygen level dependent (mq-BOLD) MRI. Next, mean parameter values were extracted from volumes-of-interest (VOIs) inside and outside of individual watershed areas (iWSA), additionally split in GM and WM, for both hemispheres and interhemispheric differences, resulting in 96 features overall. Finally, a random forest classifier was trained on data from 24 ICAS patients and 24 healthy controls (HC) to calculate feature importance scores.

In brief, we performed pCASL to obtain CBF using a label duration of 1,800 ms and a post label delay of 2,000 ms. CVR was obtained based on single-shot EPI BH-fMRI according to Pillai et al. (Pillai and Mikulis, 2015) with five end-expiratory breath-holdings of 15 s alternating with 45 s of normal breathing. Furthermore, using a bolus injection of weight-adjusted Gd-DOTA (concentration: 0.5 mmol/mL, dose: 0.1 mmol/kg, at least 7.5 mmol per subject, flow rate 4 mL/s, injection 7.5 s after DSC imaging onset) DSC-MRI yielded TTP maps calculated as the interval between global bolus arrival time and each voxel's peak signal loss (Kaczmarz et al., 2018). Additionally, rCBV was derived from DSC data with leakage correction (Hedderich et al., 2019) and *MTT, CTH and OEC* were obtained by parametric modeling (Mouridsen et al., 2014). Finally, T2 and T2^*^ mapping by multi-echo gradient-spin echo (GRASE) and gradient echo were performed for multi-parametric quantitative blood oxygen level dependent (mq-BOLD) MRI (Hirsch et al., 2014). Relative oxygen extraction fraction *rOEF* = *R2*′*/(c*·*rCBV)* was calculated from *R2*′ = (1/T2^*^) – (1/T2) and rCBV using *c* = *4/3*·π·γ·Δχ·*B*_0_ = 317 Hz at 3 Tesla (Kaczmarz et al., 2020), resulting in eight quantitative maps of perfusion, oxygenation and microvascular parameters in total (Figure 1). For detailed information see the Supplementary material.

All MRI parameter maps were carefully screened for imaging artifacts (by JG and SK with each 5 years of experience, and CP with 25 years of experience in cerebral research). No arterial transit time artifacts were observed by careful inspection of unsmoothed CBF maps. Data from five patients and six healthy controls were excluded due to the impaired quality of more than four parameters, resulting in 24 subjects in both groups. From the final data set, about 15% of all parameter maps were excluded due to low quality.

### 2.3. Feature extraction

Feature vectors were defined by extracting MRI parameter values from gray and white matter VOIs inside and outside of individual watershed areas.

- *iWSAs* were defined based on temporal perfusion delays derived from DSC-based TTP maps as described previously (Kaczmarz et al., 2018). In short, smoothed TTP-maps were segmented by masking voxels above the 90^th^ percentile of the whole brain histogram. In addition, external/cortical watershed zones were manually included and venous blood sinuses/vessels as well as the ventricular system and choroid plexus were excluded (Kaczmarz et al., 2018).
- *GM and WM* tissue masks were defined by segmenting MPRAGE data with SPM12 using default settings. The resulting probability maps were thresholded at *p* > 0.70 (Kaczmarz et al., 2021).

**Figure 2:**
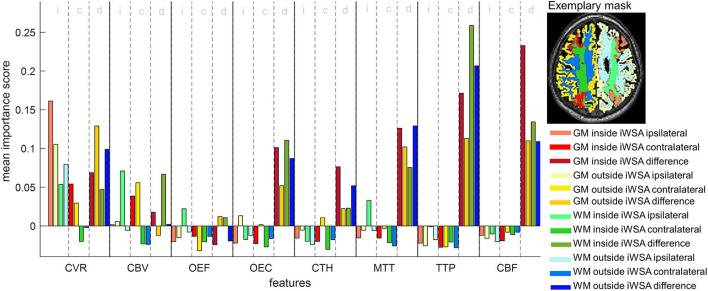
Feature importance scores of a random forest classifier trained with 96 features. Scores were averaged 1,000 times. Features were extracted from eight MRI parameter maps [cerebrovascular reactivity (CVR), cerebral blood volume (CBV), oxygen extraction fraction (OEF), oxygen extraction capacity (OEC), capillary transit time heterogeneity (CTH), mean transit time (MTT), time to peak (TTP) and cerebral blood flow (CBF)], with each sampled from 12 different VOIs that are shown as color overlays in the inset on the right. For each parameter, the first, second and third group of four bars refer to ipsilateral (i) and contralateral (c) mean values, and interhemispheric differences (d) between mean values, respectively. For each group, the color scheme is similar for GM inside of iWSAs (red), GM outside of iWSAs (yellow), WM inside of iWSAs (green) and WM outside of iWSAs (blue). The color intensity increases from group one to three.

For each subject, mean parameter values were calculated separately for hemispheres ipsilateral and contralateral to the stenosis from VOIs inside and outside of iWSAs that were split in GM- and WM-VOIs (see insert in Figure 2 for exemplary masks). Group average values of ICAS patients and healthy controls are shown in Table 1. Moreover, differences in mean parameter values between both hemispheres were extracted, resulting in 12 features per parameter (four VOIs from each hemisphere plus four interhemispheric differences) and 96 features in total (Figure 1). Furthermore, mean parameter values were calculated within whole GM and WM masks, without segmentation of iWSAs, for each hemisphere and interhemispheric differences.

**Table 1:** Group average parameter values for ICAS patients and healthy controls for all evaluated VOIs.

		**ICAS patients (*n* = 24)**				**Healthy controls (*n* = 24)**			
		**GM**		**WM**		**GM**		**WM**	
		**Inside**	**Outside**	**Inside**	**Outside**	**Inside**	**Outside**	**Inside**	**Outside**
CVR [β]	i	20.2 ± 6.2	23.7 ± 5.3	12.0 ± 4.8	16.5 ± 4.5	29.3 ± 5.8	31.1 ± 5.0	16.0 ± 3.1	21.3 ± 3.7
	c	23.0 ± 6.5	25.1 ± 5.2	15.3 ± 5.0	18.3 ± 4.8	29.1 ± 5.8	29.0 ± 5.5	16.9 ± 4.7	20.2 ± 4.9
CBV [%]	i	4.43 ± 0.36	5.13 ± 0.42	2.62 ± 0.16	3.18 ± 0.14	4.57 ± 0.45	5.26 ± 0.26	2.47 ± 0.08	3.24 ± 0.14
	c	4.23 ± 0.38	4.98 ± 0.37	2.47 ± 0.10	3.08 ± 0.15	4.59 ± 0.37	5.20 ± 0.26	2.47 ± 0.08	3.15 ± 0.10
OEF	i	0.56 ± 0.06	0.62 ± 0.05	0.94 ± 0.10	0.92 ± 0.09	0.58 ± 0.08	0.64 ± 0.06	1.02 ± 0.10	0.95 ± 0.08
	c	0.57 ± 0.06	0.63 ± 0.06	0.98 ± 0.09	0.93 ± 0.08	0.58 ± 0.05	0.63 ± 0.06	1.00 ± 0.09	0.95 ± 0.07
OEC	i	0.38 ± 0.08	0.33 ± 0.09	0.44 ± 0.07	0.37 ± 0.08	0.38 ± 0.07	0.35 ± 0.07	0.44 ± 0.07	0.38 ± 0.07
	c	0.34 ± 0.10	0.30 ± 0.09	0.39 ± 0.09	0.33 ± 0.10	0.38 ± 0.07	0.33 ± 0.08	0.42 ± 0.07	0.36 ± 0.08
CTH [s]	i	3.03 ± 1.34	2.86 ± 1.25	3.81 ± 1.70	3.17 ± 1.33	2.81 ± 0.95	2.88 ± 0.93	3.48 ± 1.11	3.09 ± 1.04
	c	2.54 ± 1.23	2.45 ± 1.21	3.25 ± 1.44	2.70 ± 1.24	2.75 ± 0.94	2.75 ± 0.93	3.41 ± 1.20	2.94 ± 1.04
MTT [s]	i	2.54 ± 0.92	2.28 ± 0.92	3.17 ± 1.01	2.57 ± 0.91	2.46 ± 0.71	2.34 ± 0.68	3.04 ± 0.81	2.54 ± 0.75
	c	2.12 ± 0.92	1.93 ± 0.90	2.69 ± 1.03	2.17 ± 0.92	2.39 ± 0.68	2.21 ± 0.69	2.93 ± 0.84	2.40 ± 0.77
TTP [s]	i	12.3 ± 1.3	11.7 ± 1.4	12.8 ± 1.4	12.1 ± 1.3	12.1 ± 1.3	11.6 ± 1.3	12.6 ± 1.4	11.9 ± 1.3
	c	11.7 ± 1.4	11.2 ± 1.3	12.3 ± 1.3	11.6 ± 1.3	12.0 ± 1.4	11.5 ± 1.3	12.6 ± 1.4	11.8 ± 1.4
CBF [ml/ 100g/min]	i	25.3 ± 6.9	25.6 ± 5.9	17.5 ± 5.3	23.6 ± 5.6	27.3 ± 6.1	26.6 ± 5.1	19.3 ± 4.7	24.6 ± 4.8
	c	30.3 ± 6.8	28.8 ± 6.0	22.1 ± 5.5	27.2 ± 5.8	28.0 ± 6.5	27.8 ± 5.3	20.1 ± 5.1	25.7 ± 5.3

Group average parameter values and standard deviations per volume-of-interest [gray matter (GM) and white matter (WM), each split in volumes inside and outside of individual watershed areas for ipsilateral (i) and contralateral (c) hemisphere], are shown for ICAS patients and healthy controls. Investigated parameters are cerebrovascular reactivity (CVR), cerebral blood volume (CBV), oxygen extraction fraction (OEF), oxygen extraction capacity (OEC), capillary transit time heterogeneity (CTH), mean transit time (MTT), time to peak (TTP) and cerebral blood flow (CBF).

### 2.4. Random forest model construction

All machine learning procedures were implemented using MATLAB's “statistics and machine learning toolbox” (MATLAB R2020a, MathWorks, Natick, MA, USA). Reported classifiers consist of 300 bootstrapped trees built by MATLAB's Treebagger function. After testing various numbers of trees in a range between 200 and 1,000 trees, 300 trees were considered the best tradeoff between increasing computing costs and improving the classifier's performance. The diversity of the trees, which reduces overfitting, was obtained by building the trees on randomly drawn subsets of subjects with replacement. The subjects that were not sampled to build a specific tree are referred to as out-of-bag observations (Breiman, 1996). At each decision split, a random subset of features was used, where the subset's size equaled the square root of the total number of features. To predict the class of a given test sample, i.e., healthy subject or ICAS patient, votes of all trees of the random forest were democratically combined and the majority determined the final prediction (see Breiman, 2001 for more details). Surrogate splits were used to handle missing data points. In case of a missing value, observations were sent to the left or right child node by choosing a surrogate variable that is most suitable to mimic the original split (Breiman et al., 1983).

### 2.5. Calculation of feature importance scores

Out-of-bag observations, i.e., data that were not used to build a specific decision tree, were employed to estimate the importance of the individual features. To this end, the out-of-bag cases first served as a test set to compute the prediction error of every tree in the ensemble. Next, all values of the feature of interest were permuted across the out-of-bag samples and the calculations were repeated. The increase in prediction error was averaged over all trees in the forest and divided by the standard deviation. The resulting measure is referred to as a feature importance score (MathWorks, Statistic and machine learning toolbox). High values indicate a high impact of the given variable on the classifier's decisions (Breiman, 1996). In this study, feature importance scores of a model trained on 96 features from inside and outside iWSAs were calculated. The whole procedure was repeated 1,000 times to gain more reliable approximations.

Due to complex interactions between features, importance scores are influenced by other features within the data set (Breiman, 2001). For this reason, recursive feature elimination (Granitto et al., 2006; Gregorutti et al., 2017) was implemented to further evaluate the ranking order, i.e., the whole feature set was repeatedly reduced by 10% of the lowest ranked features until all remaining features had an importance score > 0.1. From the resulting feature set, only the highest ranked VOI and/or VOI difference per parameter was included in the final model to avoid adding redundant features. Importance scores of the final feature set were averaged 1,000 times. Additionally, importance scores of models trained on random subsets of 12 features were calculated to validate the ranking order.

### 2.6. Validation of classification models

In this study, four classification models were investigated to evaluate the influence of VOI definition as well as feature selection. To assess the classification performances, 10-fold cross-validated models were built. To this end, all subjects were randomly assigned to one of 10 almost equally sized subsets referred to as folds. Sequentially, each fold served as test set once, while the remaining folds formed the training set. As measures of performance, the accuracy and receiver operating characteristics area under the curve (AUC) were calculated. During the tuning process various numbers of folds were tested. Ten-fold cross-validation which is common in neuroimaging applications (Lebedev et al., 2014; Jollans et al., 2019) achieved the best performance scores and was, therefore, applied to the final model.

#### 2.6.1. Influence of VOI definition

To evaluate the impact of splitting global GM and WM VOIs into VOIs considering iWSAs, a classifier based on 96 features derived from inside and outside of iWSAs was compared to a classifier based on 48 features derived from whole GM and WM hemispheres, neglecting iWSAs. Since the bisected number of features may influence the performance score, a third classifier based on an equally sized feature set, i.e., 48 features from only inside of iWSAs, was investigated. Cross-validated accuracies and AUCs were calculated for each model.

#### 2.6.2. Influence of clinical features

Adding not image-based features may improve the classifier's performance further. Therefore, the set of 96 features was extended by clinical characteristics that were significantly different between ICAS patients and healthy controls. An additional classifier was trained on the resulting feature set.

#### 2.6.3. Influence of nested feature selection

Since correlated and non-informative features can lower the classifier's performance, a model with embedded feature selection was implemented (Gregorutti et al., 2017) and compared to the model sampling all 96 features without selection. A nested approach (Jollans et al., 2019; Zhong et al., 2020) was used to avoid a feature selection bias. Based on the out-of-bag feature importance scores of the full set of 96 features, the highest ranked VOI and VOI difference per parameter was selected in an inner loop within 10-fold cross validation to this end. Next, the sampled features were sorted in order of decreasing feature importance scores. Subsequently, 12 models were trained using an increasing number of features of the resulting feature subset, starting with a model built only on the highest ranked feature, and adding features in order of decreasing importance scores. The classifiers' performances were assessed by means of the external validation set. The whole process was repeated using each cross-validation fold as test set once.

To gain a more precise approximation of the models' performances, measures were averaged by rerunning the entire algorithms 100 times.

### 2.7. Cognitive evaluation of correctly and misclassified patients

Two subgroups were formed based on each patient's likelihood of misclassification. To this end, a model including all 96 features was trained 1,000 times. Per repetition, actual and predicted class of each subject were compared. The percentage of misclassifications of the ICAS patients was calculated by dividing the number of incorrect class predictions by the number of repetitions. The two patient subgroups were then formed based on misclassification probabilities that were thresholded at 50%. The cognitive performance of both groups was evaluated.

Cognitive data of two ICAS patients were missing (one due to visual impairments, one due to lack of motivation to finish the test), which were therefore excluded from this subanalysis, resulting in 22 subjects in total.

### 2.8. Statistical analysis

Statistical analyses were carried out using MATLAB's “statistics and machine learning toolbox” (MATLAB R2020a, MathWorks, Natick, MA, USA).

A two-tailed independent sample *t-*test was used for parametric data, Mann–Whitney/Wilcoxon U statistics for non-parametric data and Pearson's chi-squared test for categorical data. We used a fast implementation of DeLong's test (DeLong et al., 1988), developed by Sun and Xu (Sun and Xu, 2014), to compare ROC curves of models trained on different feature subsets. Since there is no unbiased estimator of variance due to the overlap of training sets within cross-validation, repeated accuracies were not tested for significant differences (Bengio and Grandvalet, 2004). A threshold of α = 0.05 was used to determine statistical significance.

## 3. Results

### 3.1. Demographics

Table 2 shows demographic and clinical characteristics of ICAS patients and healthy controls, including co-morbidities, cardiovascular risk factors and medication, as well as cognitive and affective functions. Increased systolic blood pressures, antihypertensive medications, statins and antiplatelets were significantly more prevalent in the patient group, whereas the remaining characteristics including cognitive scores did not differ significantly.

**Table 2:** Clinical characteristics of ICAS patients and healthy controls.

	**ICAS patients (*n* = 24)**	**Healthy controls (*n* = 24)**	***p*-value**
Age (yrs.)	70.6 ± 6.4	70.4 ± 4.6	0.90
Female gender (no.) (%)	9 (37.5)	15 (39)	0.08
Stenotic degree (% NASCET crit.)	80.2 ± 8.9	-	-
No. right-/left-sided stenosis	16/8	-	-
Body mass index	26.8 ± 4.9	26.2 ± 4.0	0.65
Hypertension (no.) (%)	19 (79)	13 (54)	0.07
Mean BP (mmHg, sys./dias.)	154.3 ± 23.4/85.6 ± 10	140.8 ± 21.5/84.2 ± 7.5	<0.05^*^/0.59
Diabetes (no.) (%)	6 (25)	2 (8)	0.12
Smoking (no.) (%)	12 (50)	7 (29)	0.14
Mean pack-years in smokers	36.9 ± 21.2	19.4 ± 17.8	0.09
Medication (no.) (%)			
Antiplatelets	23 (96)	6 (25)	<0.01^*^
Statins	16 (67)	5 (21)	<0.01^*^
Antihypertensives	18 (75)	9 (38)	0.01^*^
CHD/PAOD (no.) (%)	12 (50)	6 (25)	0.07
TMT-A (s)	45.8 ± 15.1	48.6 ± 32.9	0.63
TMT-B (s)	139.3 ± 64.5	118.5 ± 68.9	0.16
MMSE	28.3 ± 1.9	28.8 ± 1.4	0.51
BDI	10.2 ± 10.7	7.8 ± 4.9	0.35
STAI	38.9 ± 11.5	32.8 ± 8.2	0.05

Variables are represented by the mean values and standard deviations. Two-sample t-test for age, body mass index, BP, BDI and STAI. Mann-Whitney U test for TMT-A/B and MMSE. Chi-squared test for remaining group comparisons.

* Indicates significant group differences *p* ≤ 0.05. BDI, Beck's depression inventory; BP, blood pressure; CHD/PAOD, coronary heart disease or peripheral artery occlusive disease; MMSE, mini-mental state examination; STAI, state trait anxiety inventory; TMT-A/B, trail marking test A/B.

### 3.2. Feature importance ranking

**Figure 3:**
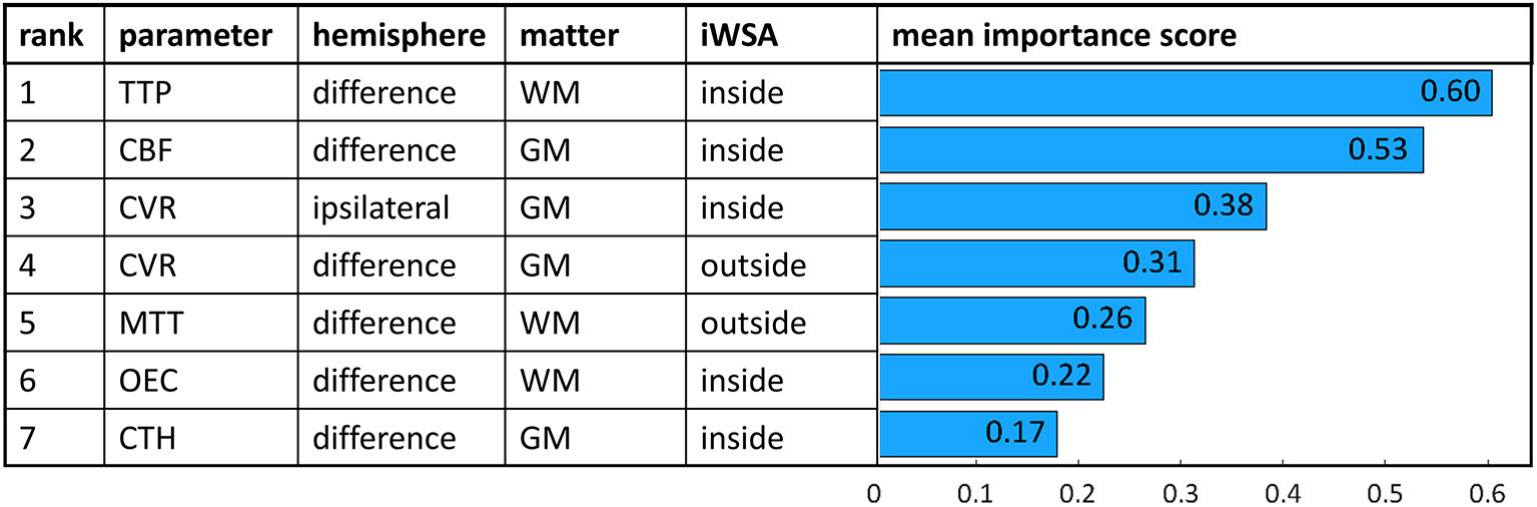
Feature importance scores of a random forest classifier trained with 7 highest ranked features. Scores were averaged 1,000 times. Corresponding parameters, VOI characteristics and mean importance scores are shown for each feature.

Importance scores for the whole set of 96 features are shown in Figure 2. Generally, interhemispheric differences of parameter values showed higher importance scores than individual hemisphere averages, except for CVR. With regard to ranking (Figure 3), the feature with the highest importance score from each group of four associated features, i.e., features derived from the same parameter in different VOIs of one hemisphere, was included. Consequently, the highest ranked features in order of decreasing importance scores are interhemispheric differences of TTP in WM, CBF in GM and ipsilateral CVR in GM, all inside of iWSAs.

Differences in the number of missing values per parameter may influence feature importance scores. CVR contained the highest number of missing measurements (14/48 subjects excluded) compared to the other parameters (TTP, MTT, OEC, CBV, CTH: 7/48 subjects excluded; CBF, OEF: 5/48 subjects excluded). However, the number of missing values per feature did not differ significantly from the mean number of missing values (for all parameters *p* > 0.08). Furthermore, no association between the importance scores and the ratio of missing values was observed.

Recursive feature elimination and feature subset evaluation confirmed the ranking order, which shows the stability of the results. After the recursive feature elimination process only the highest ranked VOI and/or VOI difference per parameter was added to the final model resulting in seven features. The mean feature importance scores of the model trained with the seven highest ranked features are shown in Figure 3. Only for MTT the highest ranked VOI was not consistent. Depending on the feature subset, MTT from GM inside of iWSAs or from WM outside of iWSAs reached a higher rank.

### 3.3. Classifier performance

We evaluated how different feature sets influence the classifier's predictive ability.

#### 3.3.1. Influence of VOI definition

First, it was investigated whether applying iWSAs improves the classifier's performance. The model trained on 48 features derived from global GM and WM VOIs gained a lower accuracy (mean ± standard deviation) of 79.0 ± 2.2% and a smaller AUC with 0.84 ± 0.04 than the model trained on 96 features from inside and outside of iWSAs (accuracy 80.1 ± 2.8%, AUC 0.86 ± 0.05). To account for the bisected number of features in the model neglecting iWSAs, it was compared to a model built on an equal number of features derived only from VOIs inside of iWSAs. The latter yielded higher scores (accuracy 81.7 ± 3.0%, AUC 0.88 ± 0.05), but Delong's test did not reveal significant differences in AUC between the two models (*p* = 0.20).

#### 3.3.2. Influence of clinical features

The systolic blood pressure and the total number of daily medications that are related to cerebrovascular disease (i.e., antiplatelets, statins, and antihypertensives) were significantly different between groups and were, therefore, added to the feature set. The resulting classifier based on 98 features achieved similar performance scores (accuracy 80.0 ± 2.9%, AUC 0.87 ± 0.02, Delong's test: *p* = 0.87) as the classifier based on 96 features. The feature importance scores of the clinical characteristics did not exceed the scores of the seven highest ranked MRI-based features.

#### 3.3.3. Influence of nested feature selection

**Figure 4:**
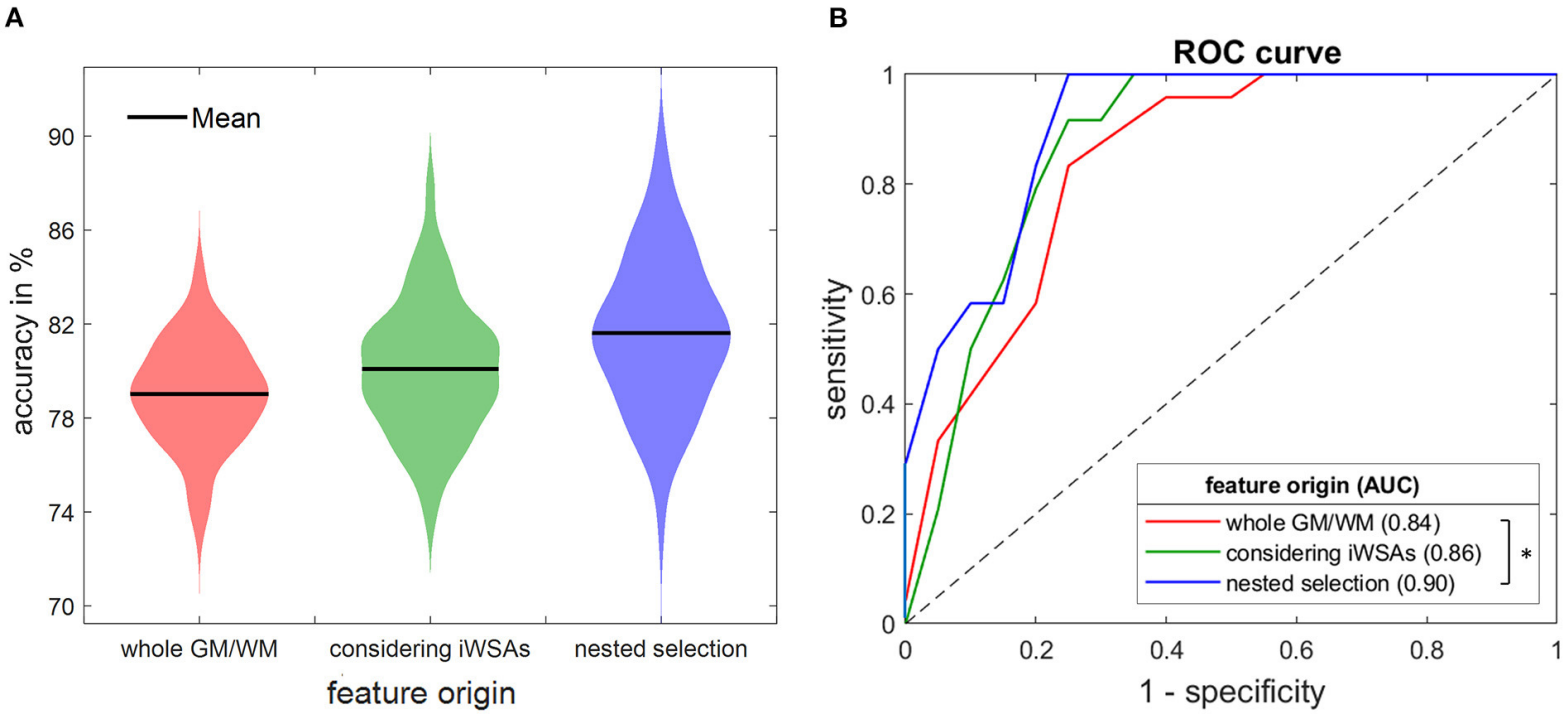
Accuracies **(A)** and ROC curves **(B)** of three random forest models. The classifier was trained on features derived from whole GM/WM VOIs (red) and from inside and outside of iWSAs without using (green) and with using nested feature selection (blue), respectively. Depicted measures were averaged 100 times. **(A)** Violin plots show mean accuracies (black line). The width of the colored area represents the proportion of classifier iterations that reached the respective accuracy. Considering iWSAs yielded higher scores. Additional feature selection improved the accuracy further. **(B)** ROC curves of the three models. AUCs differed significantly comparing the model based on whole GM/WM VOIs to the one considering iWSAs with feature selection (Delong's test, *p* < 0.05, asterisks). iWSA, individual watershed area; GM, gray matter; WM, white matter; ROC, receiver operating characteristics; AUC, area under the curve.

Additionally, nested feature selection was applied to the model trained on all 96 features, which further improved the classifier's performance. Highest performance measures were achieved by a model sampling only the six highest ranked features selected within the inner loop [accuracy 81.6 ± 3.3%, AUC 0.90 ± 0.04, sensitivity 92.8 ± 6.2 and specificity 77.9 ± 5.9 at maximum Youden's index (Youden, 1950)] (Figure 4) (Hoffmann, 2015). Adding more features did not further improve the performance, but instead resulted in a decrease of the AUC. Compared to the model trained on whole GM and WM VOIs, the model with combined improvement by splitting VOIs into iWSAs, and the use of nested feature selection achieved a significantly larger AUC (Figure 4B).

### 3.4. Impaired cognition of correctly classified ICAS patients

An association between ICAS and cognitive decline has been reported (Lal et al., 2017). For this reason, we hypothesized a superior cognitive performance in ICAS patients who were misclassified as healthy controls, compared to patients that were correctly identified by the classifier. Of the 22 ICAS-patients included in this subanalysis, five patients were misclassified in more than 70% of all repetitions, while the remaining 17 subjects received false negative predictions in <30% of the cases. Trail Making Test-B results of mostly true positive subjects were significantly higher than those of frequently misclassified subjects (152.5 ± 66.9 vs. 94.4 ± 26.1s, *p* = 0.04), indicating a poorer cognitive performance in the correctly classified subgroup. This finding was supported by trends for higher mean values of Trail Making Test-A (46.6 ± 16.7s vs. 43.4 ± 8.7s, *p* = 0.94) and lower mean values of MMSE (28.1 ± 2.0 vs. 29.3 ± 1.2, *p* = 0.32) in the mostly correctly classified group compared to the misclassified group. It should be noted however that the median scores of all subgroups were within the age adjusted normative ranges (Crum et al., 1993; Tombaugh, 2004). Age and affective function can influence cognitive abilities. However, no significant differences between the correctly and the misclassified group were found regarding age (*p* = 0.92), BDI (*p* = 0.61) or STAI (*p* = 0.50). Furthermore, more accurately predicted patients were associated with non-significantly higher NASCET scores compared to the more incorrectly predicted group (82.6 ± 8.3 vs. 76.0 ± 5.5%, *p* = 0.10). Healthy controls were not included in this evaluation since their misclassification probabilities were more homogenous with only two frequently misclassified subjects.

## 4. Discussion

In this study, we successfully applied a random forest classifier on multiparametric quantitative MRI data to predict asymptomatic, one-sided ICAS. The highest performance scores were achieved by a model with nested feature selection trained on features from inside and outside of iWSAs. Furthermore, we ranked the parameters with regard to their sensitivity to predict ICAS. Most relevant parameters in order of decreasing importance scores are interhemispheric differences of TTP and CBF followed by ipsilateral CVR.

### 4.1. Feature importance ranking

Overall, the calculated ranking order fits with previous results (Kaczmarz et al., 2021) and can be explained by the underlying pathophysiological effects of ICAS and technical limitations of the individual parameters. The high discriminative ability of interhemispheric TTP differences fits with well-known and consistently observed perfusion delays in ICAS (Nasel et al., 2001). TTP increases result from a complex interplay of reduced perfusion pressure ipsilateral to the stenosis (Baradaran and Gupta, 2020) and delayed perfusion *via* collateral flow (Schmitzer et al., 2021). MTT, which ranked in 5^th^ place, is also related to perfusion delays. However, in contrast to TTP, which can be calculated relatively easily as a descriptive parameter from the DSC time course, MTT calculation involves more sophisticated processing, i.e., deconvolution, which is more noisy and prone to error (Mouridsen et al., 2014). It therefore makes sense that MTT is ranked less important than the more robust TTP.

The second-ranked parameter is CBF derived from single-PLD pCASL, which is also well-known to be reduced ipsilateral to the stenosis (Kaczmarz et al., 2021). However, prolonged blood arrival times ipsilateral to the stenosis and in particular within iWSAs may lead to a CBF underestimation (Fan et al., 2016). Therefore, the interhemispheric CBF difference could be overestimated, resulting in higher feature importance scores. Although no artifacts were observed in our cohort, this issue could be avoided by applying time-encoded ASL in the future (van Osch et al., 2018).

CVR is the only investigated parameter that was deemed relevant twice by recursive feature elimination, i.e., as interhemispheric difference and ipsilateral. The high sensitivity is in line with previous studies, where CVR was further reported to predict stroke risk (King et al., 2011; Baradaran and Gupta, 2020). However, in our study, only third and fourth ranks were reached. This can be explained by the relatively high number of missing values compared to other parameters, which might have decreased the importance scores. This relatively high number of missing values is due to compromised quality of CVR data derived from the breath-holding task. In this respect, we expect higher reliability and data quality when hypercapnia (Pillai and Mikulis, 2015) is used. However, this requires a more complex set-up.

In general, oxygenation sensitive parameters were deemed less important than hemodynamic parameters. An explanation is the subtlety of metabolic impairments in asymptomatic ICAS patients (Baradaran and Gupta, 2020; Kaczmarz et al., 2021) who do not yet suffer from misery perfusion as defined by Baron et al. (1981).

### 4.2. Higher sensitivity of iWSAs

The highest ranked features identified by recursive feature elimination are mostly from inside of iWSAs, which is in line with previous results (Kaczmarz et al., 2021). The only exceptions are ipsilateral CVRs, which might be less reliable due to the high number of missing feature values as described above and the difference in MTT. Further evaluation of different feature sets including various MTT features revealed similar relevance of MTT VOIs inside and outside of iWSAs. However, the increased discriminative ability considering iWSAs is supported by the improved performance of the classifier trained on features from iWSAs compared to the classifier trained on global GM and WM VOIs. This also fits with previous studies on ICAS patients that proposed an increased vulnerability of water shed areas to hemodynamic impairments (Wiart et al., 2000; Nasel et al., 2001), which are associated with internal border zone infarcts (Yong et al., 2006). In particular, watershed areas were found to be highly variable and subject-specific in ICAS patients, since they shift due to individual collateral flow *via* the Circle of Willis (Kaczmarz et al., 2018). Therefore, defining iWSAs more precisely further increases sensitivity (Kaczmarz et al., 2021).

### 4.3. Increased accuracy of the nested classifier

Random forest classifiers have previously been chosen for the prediction of neurological disease based on multi-parametric MRI data because of their ability to deal with small sample sizes combined with high-dimensional correlated data (Maggipinto et al., 2017; Carlson et al., 2020). However, to address concerns regarding potential overfitting when the number of features largely exceeds the number of subjects, we built a classifier on a manually reduced feature set where similar parameters and contralateral values were excluded (data not shown). This classifier did not achieve significantly different performance scores. Nevertheless, overfitting cannot be ruled out completely. Additionally, correlated features that do not supply complementary information may decrease performance (Lebedev et al., 2014). These problems are commonly tackled by feature selection. To this end, a nested model was chosen since non-nested models lead to overoptimistic performance scores (Maggipinto et al., 2017). In this study, no performance improvement was found if selecting more than six features. A possible explanation is feature subsampling when building the individual trees, since more informative features may be neglected if correlated features, which are frequent in our data set, are added. The small number of required features for optimal performance provides benefits such as less computational cost, prevention of overfitting (Granitto et al., 2006) and shorter data acquisition times, which improves clinical applicability. For instance, MRI scans could be screened automatically for patients with an increased probability of severe ICAS, using only the most sensitive features, i.e., TTP and CBF within iWSAs.

The performance scores of our classifier suggest a similarly high predictive ability compared to other studies that applied random forest machine learning in neurological diseases (de Weerd et al., 2014; Maggipinto et al., 2017). To the best of our knowledge, there is no study that attempted prediction of asymptomatic ICAS using machine learning based on cerebral imaging data yet. However, Yin et al. (2020) used a random forest model to predict ICAS based on features contributing information on demographics, comorbidities, cardiovascular risk factors and cognition. Tested on an independent dataset the classifier reached an AUC of 0.89 and, thus, performed similar to our classifier. According to Yin et al. (2020) family history of dyslipidemia and the level of high- and low-density lipoprotein cholesterol were the most sensitive parameters to predict asymptomatic ICAS. Adding these features might further improve the performance of our classifier but this could not be tested due to lack of data. Including the systolic blood pressure and the number of medications, however, did not improve the model's performance significantly. This might be due to a limited validity of blood pressure measurements in testing situations. Additionally, blood pressure and medications are correlated which may decrease the features' importance scores as well.

### 4.4. Cognitive impairment of correctly classified patients

An association between ICAS and cognitive decline has been reported previously (Lal et al., 2017). However, not all ICAS patients are equally affected, and it cannot easily be predicted which patients will suffer from cognitive decline. Subtle damage to brain tissue caused by hemodynamic impairment has been proposed as a possible underlying pathomechanism (Balestrini et al., 2013; Lal et al., 2017). This has been supported by Buratti et al. (2016), who used ipsilateral CVR values to predict cognitive decline in asymptomatic ICAS patients. Our results support this theory, since patients that were frequently misclassified as healthy controls and are thus likely to be less affected by hemodynamic impairments showed better cognitive abilities than the correctly classified patients. The random forest classifier combines multiple parameters reflecting the patients' hemodynamic statuses in a more complex way, which may improve detection of critical hypoperfusion that may lead to cognitive impairment. However, our sample of patients with asymptomatic ICAS did not comprise subjects that suffered from severe cognitive deficits. This certainly complicates the prediction of cognitive impairment and might explain why no significant differences regarding cognitive performance were observed among ICAS patients and controls (see Table 2). Even though sensitive cognitive tests have been employed, the majority of patients showed only subtle impairments in a spatial attention task (Göttler et al., 2018), and the mean Trail making test results of all subgroups were within the age-adjusted normative range (Tombaugh, 2004). Nevertheless, especially the early detection of subtle impairments, which are not yet clinically relevant, might be crucial to prevent future decline. In a previous study, cognitive deficits have been reported to be independent of the grade of stenosis (Lal et al., 2017). This also fits our results, since the correctly classified patients showing worse cognitive performances did not have significantly higher NASCET scores than the misclassified patients. A possible explanation for this are inter-individual differences in collateral flow from the contralateral carotid artery *via* the Circle of Willis, which can compensate ipsilateral hypoperfusion (Zarrinkoob et al., 2019). Nevertheless, correctly classified patients were associated with slightly higher NASCET scores potentially caused by more pronounced hemodynamic impairments in higher grades of stenoses. However, the small sample size prohibits strong conclusions.

### 4.5. Limitations

First, the sample size in our study was relatively low, which raises concerns regarding the generalization ability of the model to other datasets. However, random forest classifiers have shown reliable accuracies and robustness to overfitting even with small sample sizes (Breiman, 2001; Jollans et al., 2019; Carlson et al., 2020). Additionally, nested feature selection was implemented to reduce the number of features and, therefore, the likelihood of overfitting. Nevertheless, overfitting cannot be excluded. Due to the limited number of observations, an external validation set was lacking, which we tried to compensate by using cross-validation, although, to validate the model further, future studies with a larger sample are urgently needed. Second, in our cohort, ICAS patients presented with more vascular risk factors than healthy controls and are thus likely to suffer from general vascular impairment. Therefore, the classification of the subjects by the random forest model might not be ICAS specific, but could be associated with other cerebrovascular diseases, which have been reported to impact cognition as well (Dichgans and Leys, 2017). Third, the association between severe hemodynamic impairments and future cognitive decline and stroke could not be evaluated and a follow-up study is required in this regard. Fourth, the classifier's performance may be vendor specific and, thus, the model may not be applicable to datasets from other scanners. The classifier's generalizability could be improved using samples from different MRI scanner vendors. Lastly, there are specific limitations regarding the individual MRI parameters, which have been discussed in detail before (Kaczmarz et al., 2021).

## 5. Conclusion

In this study, we applied a random forest classifier on multiparametric MRI data from ICAS patients and healthy controls. TTP, CBF and CVR from inside of iWSAs were identified as the most sensitive parameters to detect ICAS patients. Furthermore, using VOIs from inside of iWSAs and nested feature selection both increased the prediction accuracy of the classifier. Future extension of our approach to early detection of ICAS subgroups who suffer from severe hemodynamic impairments and who are at increased risk of cognitive deficits may improve the selection of patients who benefit from more aggressive therapy.

## Data availability statement

The raw data supporting the conclusions of this article will be made available by the authors on request.

## Ethics statement

The studies involving human participants were reviewed and approved by the Medical Ethical Board of the Klinikum rechts der Isar, Technical University of Munich. The patients/participants provided their written informed consent to participate in this study.

## Author contributions

JG, CP, and SK: conceptualization, data acquisition, and supervision. CG: data analysis. CG, JG, SK, and CP: writing. BW, MK, LS, JK, and CZ: reviewing and supervision. All authors approved the final version of the manuscript.
