# Supplementary material for: Hemodynamic MRI parameters to predict asymptomatic unilateral carotid artery stenosis with random forest machine learning
Source: Front Neuroimaging. 2023 Jan 12;1:1056503. doi: 10.3389/fnimg.2022.1056503 (PMC10406220; doi:10.3389/fnimg.2022.1056503)
Supplement: Supplementary file 1 [file Data_Sheet_1.PDF]

Supplementary Material

1. Methods

1.1 Magnetic resonance imaging

Data were acquired on a clinical 3-T Philips Ingenia MRI-Scanner (Philips Healthcare, Best, The Netherlands) using a 32- and a 16-channel head/neck-receive-coil. Imaging procedures and calculation of quantitative parameter maps have been described in detail in previous publications (Kaczmarz et al., 2021, Göttler et al., 2019).

- *pseudo-continuous arterial spin labelling* (pCASL) was performed according to the recommendations of the ISMRM perfusion study group (Alsop et al., 2015). The labelling plane was individually planned based on a phase contrast angiography of the neck. Label duration 1800 ms; post label delay 2000 ms; four background-suppression pulses; segmented 3D gradient spin echo (GraSE) readout (TE = 7.4 ms, TR = 4377 ms, α = 90°, 16 slices, TSE factor 19, EPI factor 7, acquisition voxel size 2.75 × 2.75 × 6.0 mm^3), three dynamics including a proton density weighted M0 scan; acquisition time 5:41 min.

- *breath-hold functional MRI* (BH-fMRI) was based on single-shot EPI (TE = 30 ms, TR = 1200 ms, α = 70°, 38 slices, voxel size 3 × 3 × 3 mm^3, acquisition time 5:48 min) and performed according to Pillai et al. (Pillai and Mikulis, 2015) with five end-expiratory breath-holds of 15 s altered with 45 s of normal breathing.

- *dynamic susceptibility contrast* (DSC) MRI was obtained during a bolus injection of a weight-adjusted Gd-DOTA bolus (concentration: 0.5 mmol/ml, dose: 0.1 mmol/kg, at least 7.5 mmol per subject, flow rate 4 ml/s, injection 7.5 s after DSC imaging onset) using 80 single-shot gradient-echo EPI (TE = 30 ms, TR = 1513 ms, α = 60°, FOV 224 × 224 × 100 mm^3, voxel size 2 × 2 × 3.5 mm^3, 26 slices, acquisition time 2:01 min).

- *T2-mapping* based on an eight-echo gradient spin echo (GraSE; TE_{min} = ΔTE = 16ms, TR = 8596 ms, EPI factor 47, voxel size 2 × 2 × 3 mm^3, 30 slices, acquisition time 2:23 min).

- *T2*-mapping* based on a 12-echo gradient echo (GE, TE_{min} = ΔTE = 5 ms, TR = 1950 ms, α = 30°, voxel size 2 × 2 × 3 mm^3, 30 slices, acquisition time 6:08 min).

- *Structural imaging* involved MPRAGE (TE = 4 ms, TR = 9 ms, α = 8°, TI = 1000 ms, shot interval 2300 ms, 170 slices, FOV 240 × 240 × 170 mm^3, voxel size 1.0 × 1.0 × 1.0 mm^3, acquisition time 5:59 min) for tissue segmentation and FLAIR (TE = 289 ms, TR = 4800 ms, α = 90°, inversion delay 1650 ms, TSE factor 167, 163 slices, FOV 250 × 250 × 183 mm^3, voxel size 1.12 × 1.12 × 1.12 mm^3, acquisition time 4:34 min) to screen for brain lesions.

1.2 Calculation of MRI parameter maps

All MR image processing procedures used custom MATLAB programs (MATLAB R2016b, MathWorks, Natick, MA, USA) and SPM12 (Wellcome Trust Centre for Neuroimaging, UCL, London, UK) and were conducted as reported previously (Kaczmarz et al., 2021, Kaczmarz et al., 2018, Göttler et al., 2019).

Eight quantitative maps of perfusion, oxygenation and microvascular parameters were obtained.

- *Quantitative CBF* was based on pCASL. Label and control images were motion corrected, averaged, and subtracted. CBF maps were calculated following (Alsop et al., 2015) and smoothed using a Full Width at Half Maximum (FWHM) Gaussian kernel of 5 mm. No arterial transit time artefacts were observed by careful visual inspection of unsmoothed CBF maps (by JG, CP, SK) nor by analysis of the spatial coefficient of variation. (Mutsaerts et al., 2017, Göttler et al., 2018)

- *Qualitative CVR* maps were deduced from motion corrected BH-fMRI (Pillai and Mikulis, 2015). After correcting for a global time delay, CVR was assessed using beta-values that were calculated by regression of a respiratory response function. (Vondrácková et al., 2016)

- *TTP* was derived from slice-timing corrected DSC-MRI. TTP maps were calculated as the interval between global bolus arrival time and each voxel's peak signal loss (Kaczmarz et al., 2018) and smoothed using an isotropic Gaussian kernel of 6-mm FWHM.

- *MTT, CTH and OEC* were obtained from DSC-MRI by means of a parametric modelling approach (Mouridsen et al., 2014).

- *rCBV* was calculated from DSC data with leakage correction and semiautomated AIF definition as reported previously (Kluge et al., 2016, Hedderich et al., 2019).

- *rOEF* was obtained by quantification of the BOLD effect using a multi-parametric approach (Hirsch et al., 2014) and an analytical relationship between R2′, venous CBV and venous oxygenation (Yablonskiy and Haacke, 1994). $rOEF = R2'/(c \cdot rCBV)$ was calculated from $R2' = (1/T2^*) - (1/T2)$ and leakage corrected rCBV using $c = 4/3 \cdot \pi \cdot \gamma \cdot \Delta\chi \cdot B_0 = 317$ Hz at 3 Tesla. (Kaczmarz et al., 2020)

2. Results

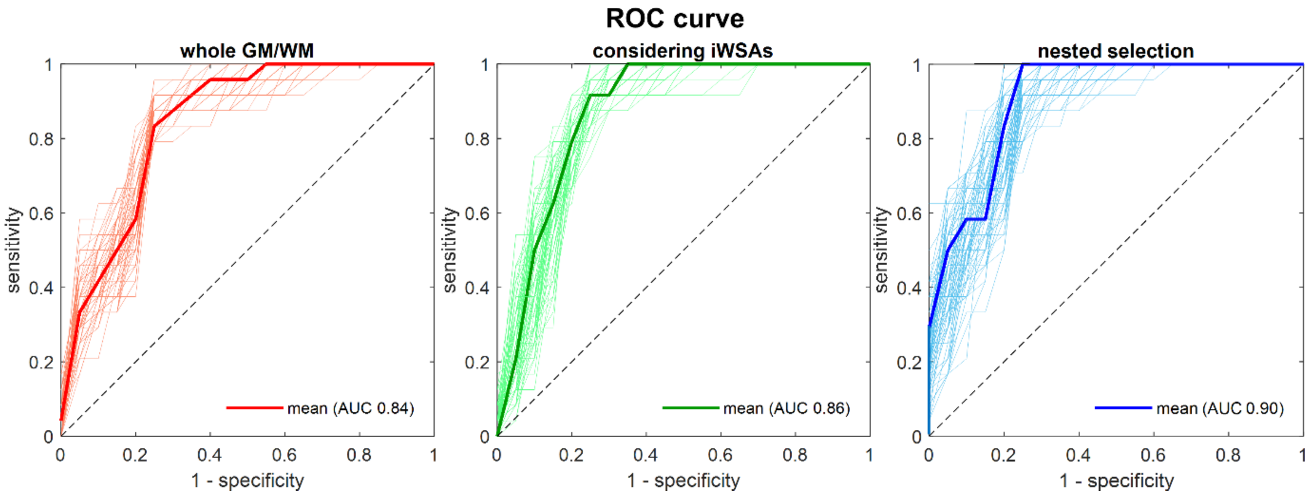

Supplementary figure 1: ROC curves of three random forest models. Features were derived from whole GM/WM VOIs (red) and from inside and outside of iWSAs without using (green) and with using nested feature selection (blue), respectively. The thin lines represent ROC curves of single iterations, and the thicker lines represent the mean ROC curve using 100 times averaged scores. Considering iWSAs yielded higher scores than whole GM/WM VOIs. Additional feature selection increased the AUC further. iWSA: individual watershed area, GM: gray matter, WM: white matter, ROC: receiver operating characteristics, AUC: area under the curve.

3. References

ALSOP, D. C., DETRE, J. A., GOLAY, X., GÜNTHER, M., HENDRIKSE, J., HERNANDEZ-GARCIA, L., LU, H., MACINTOSH, B. J., PARKES, L. M., SMITS, M., VAN OSCH, M. J., WANG, D. J., WONG, E. C. & ZAHARCHUK, G. 2015. Recommended implementation of arterial spin-labeled perfusion MRI for clinical applications: A consensus of the ISMRM perfusion study group and the European consortium for ASL in dementia. *Magn Reson Med,* 73**,** 102-16.

GÖTTLER, J., KACZMARZ, S., KALLMAYER, M., WUSTROW, I., ECKSTEIN, H., ZIMMER, C., SORG, C., PREIBISCH, C. & HYDER, F. 2019. Flow-metabolism uncoupling in patients with asymptomatic unilateral carotid artery stenosis assessed by multi-modal magnetic resonance imaging. *Journal of Cerebral Blood Flow & Metabolism,* 39**,** 2132 - 2143.

GÖTTLER, J., KACZMARZ, S., NUTTALL, R., GRIESE, V., NAPIÓRKOWSKI, N., KALLMAYER, M., WUSTROW, I., ECKSTEIN, H.-H., ZIMMER, C., PREIBISCH, C., FINKE, K. & SORG, C. 2018. The stronger one-sided relative hypoperfusion, the more pronounced ipsilateral spatial attentional bias in patients with asymptomatic carotid stenosis. *Journal of Cerebral Blood Flow & Metabolism,* 40**,** 314-327.

HEDDERICH, D., KLUGE, A., PYKA, T., ZIMMER, C., KIRSCHKE, J. S., WIESTLER, B. & PREIBISCH, C. 2019. Consistency of normalized cerebral blood volume values in glioblastoma using different leakage correction algorithms on dynamic susceptibility contrast magnetic resonance imaging data without and with preload. *Journal of Neuroradiology,* 46**,** 44-51.

HIRSCH, N., TÓTH, V., FÖRSCHLER, A., KOOIJMAN, H., ZIMMER, C. & PREIBISCH, C. 2014. Technical considerations on the validity of blood oxygenation leveŒ dependent based MR assessment of vascular deoxygenation. *NMR in Biomedicine,* 27.

KACZMARZ, S., GÖTTLER, J., PETR, J., HANSEN, M. B., MOURIDSEN, K., ZIMMER, C., HYDER, F. & PREIBISCH, C. 2021. Hemodynamic impairments within individual watershed areas in asymptomatic carotid artery stenosis by multimodal MRI. *J Cereb Blood Flow Metab,* 41**,** 380-396.

KACZMARZ, S., GÖTTLER, J., ZIMMER, C., HYDER, F. & PREIBISCH, C. 2020. Characterizing white matter fiber orientation effects on multi-parametric quantitative BOLD assessment of oxygen extraction fraction. *J Cereb Blood Flow Metab,* 40**,** 760-774.

KACZMARZ, S., GRIESE, V., PREIBISCH, C., KALLMAYER, M., HELLE, M., WUSTROW, I., PETERSEN, E. T., ECKSTEIN, H. H., ZIMMER, C., SORG, C. & GÖTTLER, J. 2018. Increased variability of watershed areas in patients with high-grade carotid stenosis. *Neuroradiology,* 60**,** 311-323.

KLUGE, A., MATHIAS, L., VIVIEN, T., THOMAS, P., CLAUS, Z. & CHRISTINE, P. 2016. Analysis of three leakage-correction methods for DSC-based measurement of relative cerebral blood volume with respect to heterogeneity in human gliomas. *Magnetic Resonance Imaging,* 34**,** 410-421.

MOURIDSEN, K., HANSEN, M. B., ØSTERGAARD, L. & JESPERSEN, S. N. 2014. Reliable Estimation of Capillary Transit Time Distributions Using DSC-MRI. *Journal of Cerebral Blood Flow & Metabolism,* 34**,** 1511-1521.

MUTSAERTS, H. J. M. M., PETR, J., VÁCLAVŮ, L., VAN DALEN, J. W., ROBERTSON, A. D., CAAN, M. W., MASELLIS, M., NEDERVEEN, A. J., RICHARD, E. & MACINTOSH, B. J. 2017. The spatial coefficient of variation in arterial spin labeling cerebral blood flow images. *Journal of Cerebral Blood Flow & Metabolism,* 37**,** 3184-3192.

PILLAI, J. J. & MIKULIS, D. J. 2015. Cerebrovascular Reactivity Mapping: An Evolving Standard for Clinical Functional Imaging. *American Journal of Neuroradiology,* 36**,** 7-13.

VONDRÁCKOVÁ, L., KRUKOWSKI, P. & J., P. 2016. Data-driven

model for evaluation of cerebrovascular-reserve measurement with hypercapnia BOLD. *Proceedings of the*

24th annual meeting of ISMRM in Singapore, 7–13 May. Abstract 3801.

YABLONSKIY, D. A. & HAACKE, E. M. 1994. Theory of NMR signal behavior in magnetically inhomogeneous tissues: The static dephasing regime. *Magnetic Resonance in Medicine,* 32**,** 749-763.
